# Burnout and its relationship with depressive symptoms in primary school teachers under the “Double Reduction” policy in China

**DOI:** 10.3389/fpubh.2024.1420452

**Published:** 2025-01-07

**Authors:** Yunhui Zhong, Shuixiu Lai, Yibo Li, Kan Yang, Hong Tang, Xiang-yang Zhang

**Affiliations:** ^1^The Third People’s Hospital of Ganzhou, Ganzhou, China; ^2^Jiangxi Environmental Engineering Vocational College, Ganzhou, China; ^3^Department of Psychiatry and Psychology, College of Basic Medical Sciences, Tianjin Medical University, Tianjin, China; ^4^Department of Psychology, Gannan Medical University, Ganzhou, China; ^5^CAS Key Laboratory of Mental Health, Institute of Psychology, Chinese Academy of Sciences, Beijing, China; ^6^Department of Psychology, University of Chinese Academy of Sciences, Beijing, China

**Keywords:** primary school teachers, Double Reduction, burnout, depression, prevalence

## Abstract

**Background:**

The “Double Reduction” policy requires schools to reduce Chinese students’ extracurricular activities and homework to lessen academic stress and improve mental well-being. However, there is limited research on primary school teachers’ psychological well-being within the context of the “Double Reduction” policy. This study examined self-reported burnout levels of primary school teachers and investigated the relationship between burnout and depressive symptoms in the context of the “Double Reduction” policy in China.

**Methods:**

A cross-sectional survey recruited 3,199 primary school teachers from 15 cities across China. The teachers’ burnout levels were assessed with the Maslach Burnout Inventory, and depressive symptoms were evaluated with the Patient Health Questionnaire depression scale.

**Results:**

Under the “Double Reduction” policy in China, 66.6% of the primary school teachers experience burnout. Individual and work-related characteristics were independently correlated with burnout. These factors included holding a bachelor’s degree (OR = 2.244, 95% CI: 1.559–3.230, *p* < 0.001), being married (OR = 0.598, 95% CI: 0.443–0.807, *p* < 0.001), being dissatisfied with one’s income (OR = 2.602, 95% CI: 2.191–3.090, *p* < 0.001), and having an intermediate professional title (OR = 1.351, 95% CI: 1.086–1.681, *p* = 0.007). The correlation coefficients between burnout subscale scores and depressive symptoms were 0.588 for emotional exhaustion, 0.585 for cynicism, and − 0.180 for professional efficacy (all *p* < 0.001).

**Conclusion:**

Our findings indicate that the prevalence of burnout among primary school teachers in China is exceptionally high, particularly under the “Double Reduction” policy. This situation is correlated with various psychological disorders, including depression. It is crucial to urgently implement psychological interventions for primary school teachers. Specifically, psychological assistance should be targeted at educators who are bachelor degree holders, married, dissatisfied with their income, and holders of an intermediate professional title.

## Introduction

The Chinese government highly prioritizes education. Chinese children and teenagers are entitled to a nine-year free and mandatory right to schooling. In recent years, relevant authorities have been attempting to reduce academic stress to prevent children and adolescents’ physical and mental health problems. In July 2021, the General Office of the Communist Party of China Central Committee and the General Office of the State Council of the People’s Republic of China jointly published “Opinions on Further Reducing the Burden of Homework and Off-Campus Training on Students in Compulsory Education” (hereafter referred to as the “Opinions”), which outlines a policy known as the “Double Reduction.” The Chinese government has mandated schools to reduce the amount of homework given to students. Additionally, it is currently prohibited for off-campus training institutions to schedule subject-based training during national holidays, rest days, and winter and summer vacations (1). The release of the “Opinions” is intended to enhance the primary role of school education, intensify oversight of off-campus training institutions, alleviate parental anxiety, prevent infringements on public interests, establish a secure educational environment, and promote the holistic development and healthy growth of students (2).

Prior research in the field of education has considered the implementation of the “Double Reduction” strategy from various perspectives and discovered a series of findings, for instance, innovating the after-school service mode; enhancing the guarantee of after-school services; improving primary and secondary school homework to meet the personalized needs of students (3); and improving the psychology of public education (4). A study investigated the changing patterns of depressive and anxiety symptoms among 28,398 Chinese elementary and junior high school students before and after the implementation of the “Double Reduction” policy. The study found a significant decrease in overall levels of depression and anxiety following the implementation of the “Double Reduction” policy (5). Meanwhile, after the release of the “Double Reduction” policy, parents have begun to pay attention to student’s physical and mental health, besides academic performance (6). Researchers have mostly analyzed the implementation strategies and effects of the “Double Reduction” policy from students’ and parents’ perspectives, while neglecting the important role and status of teachers as participants in the “Double Reduction” policy. This oversight has led to certain research limitations. Only a study utilizing grounded theory found that in-service primary and junior high school teachers exhibited differing understandings of the “Double Reduction” policy, which had a certain impact on the development of their anxiety levels. Teachers are susceptible to occupational anxiety due to the rapid and intricate nature of educational reform (7).

Burnout refers to emotional, attitudinal, and behavioral exhaustion that arises when an individual is unable to effectively cope with prolonged workplace pressures. It consists of three components: emotional exhaustion, cynicism, and reduced professional efficacy (8, 9). Burnout is recognized as an occupational disease and included in the international classification of diseases by the World Health Organization (WHO) (10). Teachers are increasingly vulnerable to job burnout, a phenomenon that has recently garnered heightened attention in academic circles (11, 12). Meta-analyses have shown that the prevalence of burnout among teachers is estimated to be approximately 50% or higher (13), which is more than twice the rate of that of healthcare workers (14, 15). The nature of teachers’ work places excessive demands on their energy, strength, and resources (16). Mild burnout is characterized by transient irritability, fatigue, worry, or frustration. Moderate burnout presents with similar symptoms but persists for a minimum of 2 weeks, while severe burnout may also manifest as physical ailments such as ulcers, chronic back pain, and migraine headaches (12).

The implementation of the “Double reduction” policy has negatively impacted teachers’ mental well-being. However, no research has examined the prevalence of primary school teachers’ burnout and its associated factors under the “Double Reduction” policy. The aim of this study was to: (1) investigate the prevalence of burnout among primary school teachers in China within the context of the “Double Reduction” policy; (2) identify the individual and job-related factors contributing to burnout in this population, and (3) examine the correlation between burnout and depressive symptoms.

## Methods

### Study design and participants

This study was a cross-sectional survey aimed at evaluating the job burnout and other psychological conditions of primary school teachers in China within the context of the “Double Reduction” policy. An online questionnaire was developed and distributed via WeChat, one of mainland China’s most important social media platforms. The data was collected between September 14 and October 31, 2022, and 3,199 primary school teachers from 15 cities across the country participated in the survey.

The study was approved by the Institutional Review Board (IRB) of the Third People’s Hospital of Ganzhou. Each participant was required to sign an electronic informed consent form prior to completing the survey. The information of all participants was kept confidential.

### Measures and procedures

#### Basic information

Demographic and work-related information was collected, including age, sex, height, weight, educational background, fertility situation (no/one/two/three children), years of teaching, income satisfaction (yes/no), marital status (single, married, divorced, or widowed), type of school (private/public), location of school (urban/rural), class adviser (yes/no), and professional qualifications.

#### Burnout

The Chinese version of the Maslach Burnout Inventory-General Survey (MBI-GS) (17, 18), a widely used measure among teachers in China, was utilized to evaluate job burnout. The MBI-GS has demonstrated satisfactory reliability and validity (19). It comprises 15 items and measures three dimensions of occupational burnout. Emotional exhaustion (EE) refers to the state of being emotionally drained or depleted due to work-related stress; cynicism (CY) is defined as having negative or cynical attitudes toward work; and professional efficacy (PE) is defined as a positive sense of accomplishment and success in the workplace. Each item was evaluated using a 7-point frequency range scale (0 = “never” to 6 = “daily”). The overall score of each subscale was divided into three tertiles: high, moderate, and low. According to previous large sample studies of the Chinese population (20, 21), the cutoffs for each tertile of burnout were determined as follows: low EE < 9, moderate EE = 9–13, high EE > 13; low CY < 3, moderate CY = 3–9; high CY > 9; low PE > 30, moderate PE = 30–18, high PE < 18. A score in the highest tertiles of EE, in combination with the highest tertiles of CY or the lowest tertiles of PE indicates burnout syndrome, according to the “exhaustion +1” criterion (22). Due to the considerable variation in the literature regarding the definition of burnout, an alternative formula was used to calculate the prevalence of burnout. This restrictive definition combines a high EE and high CY with a low PE subscale score (23).

#### Depression

The Patient Health Questionnaire-9 (PHQ-9) was used to detect depressive symptoms (24). The participants were instructed to evaluate their emotions over the previous 14 days using a 4-point Likert scale, ranging from 0 = “not at all” to 3 = “nearly every day.” It is widely employed in research due to its high yield (validity and reliability) in screening depressive symptoms, particularly in non-clinical settings compared to other recognized depression-screening questionnaires (25). The Cronbach’s *α* coefficient of the PHQ-9 was 0.92.

#### Statistical analysis

The participants’ demographic and work-related variables were compared between the burnout and non-burnout groups using the chi-square and independent sample T tests. The binary logistic regression model was utilized to identify factors independently associated with the experience of burnout. Subsequently, to identify the independent factors associated with MBI-GS scores, we employed stepwise multivariate linear regression models, with the MBI-GS subscores as dependent variables. Other variables with a potential correlation with MBI-GS subscale scores and PHQ-9 scores were examined with the Pearson correlation analysis and a linear regression model. Bonferroni corrections were implemented to account for multiple testing. A two-tailed test at *p* < 0.05 was set to be statistically significant. All statistical analyses were performed using SPSS (version 26.0).

## Results

### Demographic characteristics

The male participants totaled 1,596 (49.9%) and the females were 1,603 (50.1%). The respondents’ ages ranged from 22 to 69 years, with an average age of 39.51 ± 10.37 years. The average body mass index (BMI) was 23.23 ± 6.81 kg/m2. Table 1 presents more detailed information about participants’ demographic and job-related characteristics.

**Table 1:** Demographic data of participants with and without burnout.

Variable	Total	Non-burnout	Burnout	*X*^2^	*p*-value
	*N* = 3,199	(*n* = 282, 39.94%)	(*n* = 424, 60.06%)		
Sex					
Male	1,596 (49.9%)	514 (48.1%)	1,082 (50.8%)	1.994	0.158
Female	1,603 (50.1%)	554 (51.9%)	1,049 (49.2%)		
Education					
Junior college or less	620 (19.4%)	245 (22.9%)	375 (17.7%)	35.239	**<0.001**
Bachelor	1985 (62.1%)	681 (63.8%)	1,304 (61.4%)		
Master or above	594 (18.5%)	142 (13.3%)	452 (20.9%)		
Marital status					
Never married	440 (13.8%)	142 (13.3%)	298 (13.9%)	7.521	**0.023**
Married	2,545 (79.6%)	872 (81.6%)	1,673 (78.5%)		
Divorced or Widowed	214 (6.6%)	54 (5.1%)	160 (7.6%)		
Fertility situation					
None	563 (17.6%)	175 (16.4%)	388 (18.2%)	4.848	0.183
One child	1,492 (46.6%)	484 (45.3%)	1,008 (47.3%)		
Two children	968 (30.3%)	347 (32.5%)	621 (29.1%)		
three children	176 (5.5%)	62 (5.8%)	114 (5.4%)		
Income satisfaction					
Yes	1,245 (38.9%)	543 (50.8%)	702 (32.9%)	95.896	**<0.001**
No	1954 (61.1%)	525 (49.2%)	1,429 (67.1%)		
Residence of school					
Urban	1,202 (37.6%)	356 (33.3%)	846 (39.7%)	12.293	**<0.001**
Rural	1997 (62.4%)	712 (66.7%)	1,285 (60.3%)		
Teacher in charge of class					
Yes	1,589 (49.7%)	525 (49.2%)	1,064 (49.9%)	0.170	0.68
No	1,610 (50.3%)	543 (50.8%)	1,067 (50.1%)		
Professional qualifications					
Junior professional title	1,143 (35.7%)	417 (39.0%)	726 (34.1%)	7.699	**0.021**
Intermediate professional title	1,675 (52.4%)	529 (49.5%)	1,146 (53.8%)		
Senior professional title	381 (11.9%)	122 (11.5%)	259 (12.1%)		
BMI					
< 18.5	266 (8.3%)	91 (8.5%)	175 (8.2%)	4.563	0.102
18.5–24	1776 (55.5%)	618 (57.9%)	1,158 (54.3%)		
> 24	1,157 (36.2%)	359 (33.6%)	798 (37.5%)		
Age					
< 30	582 (18.2%)	218 (20.4%)	364 (17.1%)	23.953	**<0.001**
30–40	1,416 (44.3%)	408 (38.2%)	1,008 (47.3%)		
> 40	1,201 (37.5%)	442 (41.4%)	759 (35.6%)		
Years of teaching					
< 6 years	586 (18.3%)	212 (19.8%)	374 (17.6%)	26.188	**<0.001**
6–10 years	430 (13.4%)	132 (12.3%)	298 (13.9%)		
11–20 years	1,005 (31.4%)	281 (26.3%)	724 (33.9%)		
> 20 years	1,178 (36.9%)	443 (41.6%)	735 (34.6%)		

Values expressed as no. (%) or mean (±standard deviation). Bold values were considered to have scientific significance.

### Prevalence of burnout in primary school teachers

Burnout is defined as a combination of high EE coupled with high depersonalization (CY) or low personal accomplishment (PE) subscale scores. During the implementation of the “Double Reduction” policy, the prevalence of burnout in Chinese primary school teachers was 66.6% (2,131/3,199, 95% CI = 64.98–68.25%). The prevalence estimate of male respondents’ burnout was 67.79% (1,082/1,596), and that of females was 65.44% (1,049/1,603). For each component of burnout, the prevalence of EE, CY, and PE was 63.39, 39.42, and 21.38%, respectively.

Chi-square tests also indicated significant differences between the burnout and non-burnout groups in terms of education, marital status, income satisfaction, school residence, professional qualifications, age, and years of teaching (all *p* < 0.05). The burnout rates of each type of variable are shown in Table 1. Specifically, those who had severe burnout comprised 30–40-year old primary school teachers with more than 20 years teaching experience, who were dissatisfied with their income, taught in rural schools, had a bachelor’s degree, were married, and had an intermediate professional title. No significant differences were found, in terms of sex, fertility status, teacher in charge of class, and BMI (all *p* > 0.05), between the burnout and non-burnout groups.

Moreover, the binary logistic regression model indicated that the following variables were independently correlated with burnout: bachelor’s degree holder (OR = 2.244, 95% CI: 1.559–3.230, *p* < 0.001), married (OR = 0.598, 95% CI: 0.443–0.807, *p* < 0.001), dissatisfaction with income (OR = 2.602, 95% CI: 2.191–3.090, *p* < 0.001), intermediate professional title (OR = 1.351, 95% CI: 1.086–1.681, *p* = 0.007).

### Factors associated with burnout and its three components in primary school teachers

The average burnout score was 17.305 ± 7.08 on the EE subscale, 10.905 ± 5.52 on the CY subscale, and 27.781 ± 6.65 on the PE subscale. The MBI-GS subscale scores, grouped according to demographics and work-related variables, are presented in Table 2. Multiple linear regressions were conducted to identify independent factors associated with each MBI-GS subscore. EE was significantly associated with education (*β* = 1.498, t = 5.591, *p* < 0.001), dissatisfaction with income (*β* = 3.974, t = 15.686, *p* < 0.001), being in charge of a class (*β* = 0.710, *t* = 2.936, *p* = 0.003), and professional qualifications (*β* = 0.584, *t* = 2.284, *p* = 0.022). CY was independently correlated with education (*β* = 1.865, *t* = 9.040, *p* < 0.001), dissatisfaction with income (*β* = 2.525, *t* = 12.870, *p* < 0.001), residence of school (*β* = 0.694, *t* = 3.054, *p* = 0.002), professional qualifications (*β* = 0.733, *t* = 3.744, *p* < 0.001), and sex (*β* = 1.089, *t* = 5.081, *p* < 0.001). PE was independently correlated with dissatisfaction with income (*β* = 1.501, *t* = 6.033, *p* < 0.001), residence of school (*β* = 1.324, *t* = 4.593, *p* < 0.001), age (*β* = 0.979, *t* = 2.958, *p* = 0.003), and fertility situation (*β* = 0.391, *t* = 2.453, *p* = 0.014). Altogether, the study revealed that dissatisfaction with income was independently associated with all dimensions of burnout.

**Table 2:** MBI-GS subscale scores in grouped demographics and work-related variables.

Variable	EE	CY	PE
Sex			
Male	17.34 ± 7.13	11.23 ± 5.62	28.02 ± 6.63
Female	17.26 ± 7.03	10.58 ± 5.39	27.54 ± 6.65
*F*	0.09	11.148***	4.273*
Education			
Junior college or less	16.15 ± 6.82	9.21 ± 4.99	28.44 ± 7.41
Bachelor	17.19 ± 7.13	10.45 ± 5.27	27.66 ± 6.83
Master or above	18.91 ± 6.89	14.20 ± 5.55	27.49 ± 4.89
*F*	24.268***	155.817***	3.960*
Marital status			
Never married	17.14 ± 7.36	11.16 ± 5.59	26.88 ± 6.18
Married	17.25 ± 7.10	10.73 ± 5.55	27.83 ± 6.75
Divorced or Widowed	18.25 ± 6.14	12.44 ± 4.63	29.07 ± 6.00
*F*	2.087	10.096***	8.158***
Fertility situation			
None	17.14 ± 7.10	11.03 ± 5.54	27.23 ± 6.45
One child	17.43 ± 7.09	10.70 ± 5.55	28.31 ± 6.81
Two children	17.02 ± 6.85	10.81 ± 5.27	27.54 ± 6.64
Three children	18.34 ± 7.80	12.74 ± 6.16	26.41 ± 5.37
*F*	2.052	7.369***	7.350***
Income satisfaction			
Yes	15.03 ± 5.60	9.66 ± 4.83	28.81 ± 6.42
No	18.75 ± 7.33	11.70 ± 5.78	27.12 ± 6.70
*F*	224.721***	107.264***	49.892***
Residence of school			
Urban	17.77 ± 6.55	12.14 ± 5.29	27.13 ± 5.71
Rural	17.03 ± 7.37	10.16 ± 5.51	28.18 ± 7.12
*F*	8.271**	99.041***	18.801***
Teacher in charge of class			
Yes	17.67 ± 7.27	11.09 ± 5.60	27.73 ± 6.57
No	16.94 ± 6.87	10.73 ± 5.43	27.83 ± 6.71
*F*	8.391**	3.421	0.211
Professional qualifications			
Junior professional title	16.70 ± 6.73	10.45 ± 5.22	27.23 ± 6.69
Intermediate professional title	18.13 ± 7.48	11.28 ± 5.92	27.89 ± 6.72
Senior professional title	15.49 ± 5.62	10.60 ± 4.32	28.93 ± 5.99
*F*	28.366***	8.469***	9.946***
BMI			
< 18.5	17.95 ± 7.60	11.63 ± 6.54	27.69 ± 5.94
18.5–24	16.91 ± 7.02	10.43 ± 5.30	27.94 ± 7.00
> 24	17.76 ± 7.01	11.46 ± 5.52	27.55 ± 6.21
*F*	6.194**	14.921***	1.249
Age			
< 30	17.14 ± 7.13	11.03 ± 5.60	26.95 ± 6.21
30–40	18.20 ± 7.24	11.95 ± 5.76	27.45 ± 6.44
> 40	16.33 ± 6.72	9.61 ± 4.89	28.58 ± 6.99
*F*	22.996***	60.237***	15.094***
Years of teaching			
< 6 years	17.13 ± 7.47	11.15 ± 6.04	27.49 ± 6.45
6–10 years	17.77 ± 7.04	11.97 ± 5.75	27.03 ± 6.05
11–20 years	18.11 ± 6.97	11.72 ± 5.46	27.42 ± 6.43
> 20 years	16.53 ± 6.90	9.69 ± 4.97	28.51 ± 7.06
*F*	9.881***	33.130***	7.896***

*p<0.05, **p<0.01, ***p<0.001. MBI-GS, Maslach Burnout Inventory-General Survey; EE, emotional exhaustion; CY, cynicism; PE, professional efficacy.

### The association between burnout and depressive symptoms in primary school teachers

The mean score of PHQ-9 was 6.30 ± 6.60. With a cut-off score of 4, the overall prevalence of depressive symptoms in primary school teachers was 45.7%. The correlation coefficients between the score of each MBI-GS subscale and the PHQ-9 score were 0.588 for EE, 0.585 for CY, and − 0.180 for PE (all *p* < 0.001). These associations remained statistically significant following Bonferroni corrections. A stepwise multiple regression model showed that scores of EE (*β* = 0.329, *t* = 17.423), CY (*β* = 0.372, *t* = 14.965), and PE (*β* = −0.061, *t* = −4.350) were independently associated with the PHQ-9 score. Collectively, these three components of burnout explained 40% of the variance (adjusted R^2^) in the PHQ-9 (*F* = 712.64, *p* < 0.001).

## Discussion

Regardless of numerous studies on teacher burnout prior to the implementation of the “Double Reduction” policy, only a few studies have investigated burnout syndrome in the context of this policy. To the best of our knowledge, this is the first nationwide cross-sectional survey on primary school teachers’ job burnout under the “Double Reduction” policy. The main findings of this study include the following: (1) up to 66.6% of the respondents met the burnout criteria; (2) personal factors (i.e., educational background, marital status, income satisfaction) and job-related factors (professional qualifications) were associated with burnout; (3) the burnout levels were associated with the severity of depressive symptoms.

Our findings revealed an exceedingly high prevalence (66.6%) of burnout among primary school teachers, rendering them psychologically vulnerable under the “Double Reduction” policy. According to consistent findings from prior research, primary school teachers are especially susceptible to experiencing burnout. The prevalence of burnout among primary school teachers ranges from 24.5 to 54% in various studies (26–29). The substantial variations observed in various studies are not solely due to regional disparities, but they stem from different approaches used in defining burnout (30, 31). Burnout among teachers was already a problem in China before the implementation of the “Double Reduction” policy (32, 33), and our work seems to suggest that the policy may have worsened burnout. Several assumptions may account for this phenomenon. First, the “Double Reduction” policy is still in its early stages of implementation, and teachers often find it challenging to swiftly adapt to new pedagogical methods and requirements, leading to an increased workload. The introduction of novel teaching approaches, resource preparation, assessment methods, among others, may contribute to heightened stress and fatigue among teachers (34). Second, parents’ concerns about educational quality triggered by the “Double Reduction” policy may escalate their expectations of primary school teachers. Increased societal attention to education may elevate teachers’ social pressure, causing heightened anxiety and tension (7). To achieve these goals, teachers are required to innovate teaching methods, individualize lesson plans, and provide additional in-class activities to replace what was previously done outside the classroom. For instance, teachers now spend more time on designing engaging and diverse in-class learning experiences, assessing students’ learning progress, and addressing varied academic needs. These additional responsibilities significantly amplify their workload, often spilling into personal time and contributing to stress and exhaustion. Third, during periods of policy reform, teachers might perceive a threat to their professional identity. A lack of respect and acknowledgment for educators may contribute to fatigue and dissatisfaction with their work (35). Fourth, under the policy, primary school teachers are tasked not only with academic teaching but also with managing after-school services, such as extended care or extracurricular activities. This expands their roles beyond traditional classroom teaching, requiring them to act as caregivers, organizers, and even counselors. Many teachers report feeling unprepared or inadequately trained for these additional roles, leading to feelings of inefficacy and frustration (36). Altogether, theoretically, there is a notable negative association between the adoption of the “Double Reduction” policy and the incidence of burnout among primary school educators.

Our study also identified a series of factors associated with burnout among primary school teachers in China during the implementation of the “Double Reduction” policy. Sociodemographic factors, such as educational background, marital status, income satisfaction, and professional qualifications, are statistically significant predictors of burnout. We found that primary school teachers with a bachelor’s degree showed a higher rate of burnout than teachers with an education or master’s degree or those who attended junior college. A survey on the educational attainment of primary school teachers in China revealed that 78.3% of the country’s primary school teachers hold a bachelor’s degree (37). In China, teachers with a technical secondary school degree are basically engaged in administrative and logistical school work, while teachers with a graduate degree are more engaged in school curriculum development, project research, etc. Thus, primary school teachers with a bachelor’s degree become teaching staff who directly engage students. Such teaching staff encounter more parental demands and expectations, increasing their pressure and potentially resulting in professional burnout. Furthermore, compared to primary school teachers with junior and senior professional titles, those with intermediate professional titles are more inclined to suffer from professional burnout. They may be in an intermediate stage of career development and have ambiguous or unstable career prospects; this uncertainty may make them more susceptible to burnout. However, primary school teachers with junior professional titles are usually younger and more likely to be enthusiastic about their new teaching careers. Thus, they experience the lowest burnout prevalence (38). Teachers with senior professional titles usually have richer professional experience and teaching skills (39). They may be more mature and able to handle various teaching challenges more effectively. They may more easily adapt to work pressures and reduce the occurrence of burnout due to their professional maturity. In line with previous studies, we found a higher prevalence of burnout among married primary school teachers than the unmarried (40). Married primary school teachers may have more family responsibilities such as child care and family financial support. These additional responsibilities can create challenges in maintaining a work-life balance, which can lead to an increased risk of burnout. Moreover, family conflict and family stress can negatively impact a teacher’s job. Married teachers may face communication issues or other family relationship challenges with partners or family members that may also increase the risk of burnout.

Teachers’ dissatisfaction with their income was significantly correlated with every dimension of the burnout experience, which is consistent with previous research findings (41–43). For example, a study on preschool teachers revealed that those who expressed dissatisfaction with their income were more likely to experience burnout compared to their colleagues. Presumably, reduced income satisfactory would lead to a deficient working motivation, which contributes to burnout (42). Thus, income dissatisfaction may reduce the career motivation of primary school teachers. If teachers feel that their work and input are not proportional to their income, they may lose enthusiasm for their career in education, thereby increasing the risk of burnout. Moreover, due to income dissatisfaction, primary school teachers may feel that their profession is unfair, especially when compared with other professions. This feeling can trigger career dissatisfaction and lead to burnout.

Another important finding is that, under the “Double Reduction” policy, the burnout experienced by primary school teachers in China is positively correlated with depression. The latest report has demonstrated that after implementing the policy, students have fewer burdens, while teachers have more, coupled with mental health problems such as depression and anxiety (44). The bi-directional link between burnout and depression has been widely recognized in many occupational groups (12, 45, 46). With the policy’s implementation, teachers must quickly improve classroom instruction and reduce post-school assignments to alleviate students’ academic burden. They must also provide after-school services. Effective teaching requires the intelligence and enthusiasm of teachers and a significant amount of time and resources. Under the strictest burden-reduction policy ever, the workload of teachers has increased exponentially, which can easily lead to depression (44). Individuals suffering from depression frequently encounter challenges in meeting interpersonal, time-management, and productivity demands. They may also experience psychological issues, decrease in work quality, increased absence due to illness, and higher rates of work disability, all of which can contribute to burnout (47). Due to the cross-sectional design, this study did not establish a causal relationship between burnout and depression.

Our data suggest that there is no statistical association between several factors and primary school teacher job burnout. For instance, there is no statistically significant difference in the prevalence of burnout between male and female primary school teachers, which is inconsistent with previous research (48). Several studies have reported significantly higher burnout scores among female teachers compared to male teachers, particularly in terms of emotional exhaustion, depersonalization, and intellectual burnout. However, there are also other studies that have found higher levels of burnout among male teachers (12, 49). The absence of gender differences in burnout among primary school teachers could be because social perceptions and expectations of gender roles may have changed over time; thus, male and female teachers experience similar levels of burnout (50, 51). However, this study was conducted during the preliminary stages of implementing the “Double Reduction” policy. As its implementation advances, investigating gender differences in burnout in future research is necessary.

### Strengths and limitations

The major strength of this study is that it provides novel insights into the impact of the “Double Reduction” policy on primary school teachers’ psychological well-being, a topic that has received limited attention in the literature. Moreover, a large, nationally representative sample was used, which enhances the generalizability of the findings. This study has several limitations. First, this cross-sectional survey, conducted at a single time point, could not compare burnout levels before and after the “Double Reduction” policy was implemented. Furthermore, limited information was available regarding the specific factors of the policy that contribute to an increased prevalence of teachers’ burnout. Therefore, our findings provide no evidence of a causal relationship between the “Double Reduction” policy’s implementation and high levels of job burnout. Second, without a consensus regarding the diagnosis of job burnout, it is challenging to directly compare the prevalence of job burnout. A recent review revealed that the literature employs at least 47 different definitions of burnout prevalence when utilizing the MBI tool for measurement (31). Therefore, it is essential for future research to reach a consensus on the classification of different levels of job burnout. Third, the presence of burnout was examined through the use of an online self-administered questionnaire, which may underestimate or exaggerate their symptoms due to social desirability or personal interpretations of burnout, and compromise the reliability and validity of the measurement. Fourth, some modifiable factors that are potentially associated with burnout syndrome and depression in primary school teachers such as job stress and personality traits were not measured in this study.

### Practical implications

The findings highlight the urgent need for mental health support programs specifically designed for primary school teachers, especially those impacted by policy changes such as the “Double Reduction.” Interventions like stress management workshops, counseling services, and adjustments to teachers’ workloads could help alleviate burnout and depression. Future research should examine the long-term effects of the “Double Reduction” policy on both teachers and students. Additionally, further studies could explore interventions that mitigate the psychological impact of such policy reforms on educators, providing valuable insights to inform future educational policies.

## Conclusion

In summary, our findings indicate that the prevalence of burnout among primary school teachers in China is exceptionally high under the “Double Reduction” policy. This high prevalence is also associated with other psychological disorders such as depression. This has significant implications for the mental well-being of teachers, who are directly responsible for implementing the “Double Reduction” policy. Psychological interventions for primary school teachers are urgently needed. Teachers who have a bachelor’s degree, are married, dissatisfied with their income, and have an intermediate professional title should receive more psychological assistance.

## Data Availability

The raw data supporting the conclusions of this article will be made available by the authors, without undue reservation.
